# Supplementary material for: Magnetostatic interaction between Bloch point nanospheres
Source: Sci Rep. 2023 May 3;13:7171. doi: 10.1038/s41598-023-34167-y (PMC10156691; doi:10.1038/s41598-023-34167-y)
Supplement: Supplementary file 1 — Supplementary Information. [file 41598_2023_34167_MOESM1_ESM.pdf]

Supplementary Material - Magnetostatic interaction between Bloch Point nanospheres

Cristobal Zambrano-Rabanal

Departamento de Ciencias Físicas, Universidad de La Frontera, Casilla 54-D, Temuco, Chile

Boris Valderrama

Facultad de Física, Pontificia Universidad Católica de Chile, Casilla 306, Santiago, Chile

Felipe Tejo

Escuela de Ingeniería, Universidad Central de Chile,
Avda. Santa Isabel 1186, 8330601 Santiago, Chile

Ricardo Gabriel Elías

Departamento de Física, Universidad de Santiago de Chile (USACH),
Avda. Víctor Jara 3493, Santiago, Chile

Alvaro S. Nunez

Departamento de Física, FCFM, Universidad de Chile, Santiago, Chile. and
Centro de nanociencia y nanotecnología CEDENNA,
Avda. Ecuador 3493, Santiago, Chile

Vagson L. Carvalho-Santos

Departamento de Física, Universidade Federal de Viçosa, 36570-900, Viçosa, Brazil

Nicolás Vidal-Silva

Departamento de Ciencias Físicas, Universidad de La Frontera, Casilla 54-D, Temuco, Chile.

I. SUPPLEMENTARY MATERIAL

A. Magnetostatic field generated by the BP in the whole space

In this Supplementary Material, we explicitly show the calculation of the magnetostatic field generated by the BP in the whole space. We recall the definition of the demagnetizing field, given by $\mathbf{H}_d(\mathbf{r}) = -\nabla U_d(\mathbf{r})$, where

$$U_d(\mathbf{r}) = \frac{M_s}{4\pi}\left(-\int_{V'}\frac{\nabla\cdot\mathbf{m}(\mathbf{r}')}{|\mathbf{r}-\mathbf{r}'|}dV' + \int_{S'}\frac{\mathbf{m}(\mathbf{r}')\cdot\mathbf{n}'}{|\mathbf{r}-\mathbf{r}'|}dS'\right). \tag{1}$$

Additionally, the normalized magnetization field, expressed in spherical coordinates, is $\mathbf{m} = (\sin\Theta(\mathbf{r})\cos\Phi(\mathbf{r}), \sin\Theta(\mathbf{r})\sin\Phi(\mathbf{r}), \cos\Theta(\mathbf{r}))$. To describe the BP magnetic profile, we use the following ansatz already presented in the main text

$$\Theta(\theta) = p\theta + \pi(1-p)/2 \tag{2}$$

$$\Phi(\phi) = q\phi + \gamma, . \tag{3}$$

To perform our calculations, it is convenient to write the BP magnetic profile explicitly in terms of the components of the magnetization field in the spherical basis, that is

$$m_r = \cos\gamma\sin^2\theta + p\cos^2\theta \tag{4}$$

$$m_\theta = \sin\theta\cos\theta\,(\cos\gamma - p) \tag{5}$$

$$m_\phi = \sin\gamma\sin\theta. \tag{6}$$

Thus, the volumetric and superficial magnetic charges are evaluated as

$$\nabla\cdot\mathbf{m} = \frac{1}{r}\left[\left(\cos^2\theta + 1\right)\cos\gamma + p\sin^2\theta\right] \tag{7}$$

$$\mathbf{m}\cdot\mathbf{n} = \cos\gamma\sin^2\theta + p\cos^2\theta. \tag{8}$$

Additionally, due to the spherical symmetry of the nanoparticle geometry, we expand the Green's function $G(\mathbf{r},\mathbf{r}') = |\mathbf{r}-\mathbf{r}'|^{-1}$ into the spherical harmonics basis

$$\frac{1}{|\mathbf{r}-\mathbf{r}'|}=\sum_{l=1}^{\infty}\sum_{m=-l}^{l}\frac{4\pi}{2l+1}\frac{r_<^l}{r_>^{l+1}}Y_{l,m}(\theta,\phi)Y_{l,-m}(\theta',\phi'), \tag{9}$$

where $Y_{l,m}(\theta,\phi)$ is a spherical harmonic function of degree l and order m, formally defined as

$$Y_{l,m}=\sqrt{\frac{2l+1}{4\pi}\frac{(l-m)!}{(l+m)!}}P_l^m(\cos\theta)e^{im\phi}, \tag{10}$$

being $P_l^m(x)$ the associated Legendre polynomials, and $r_<(r_>)$ corresponds to the smaller (larger) value between r and r'. By substituting Eqs. (2 - 10) into Eq. (1), and after some algebraic manipulation, we obtain that the magnetostatic potential is given by

$$U_d(\mathbf{r})=\frac{M_s}{2}\sum_{l=1}^{\infty}P_l(\cos\theta)\left[\frac{r_<^l}{r_>^{l+1}}R^2\left(\frac{1}{3}(2\cos\gamma+p)\frac{2\delta_{0,l}}{2l+1}+\frac{2}{3}(p-\cos\gamma)\frac{2\delta_{2,l}}{2l+1}\right)\right.$$
$$\left.-\int_0^R\frac{r_<^l}{r_>^{l+1}}r'dr'\left(\frac{2}{3}(2\cos\gamma+p)\frac{2\delta_{0,l}}{2l+1}-\frac{2}{3}(p-\cos\gamma)\frac{2\delta_{2,l}}{2l+1}\right)\right], \tag{11}$$

where $\delta_{\alpha,\beta}$ is the Kronecker delta. In the following, we solve Eq. (11) for the regions inside and outside the sphere.

1. *Region $r \leq R$ (inside the sphere)*

Here we have to properly evaluate $r_<$ and $r_>$ in the region of interest. For the first term of Eq. (11) is evident that $r_<=r$ and $r_>=R$. However, in the second term the radial integral involved in Eq. (11) must be carefully evaluated. Indeed, it can be written in a more convenient way to explicitly recognize $r_<$ and $r_>$

$$\int_0^R\frac{r_<^l}{r_>^{l+1}}r'dr'=\frac{1}{r^{l+1}}\int_0^r r'^{l+1}dr'+r^l\int_r^R\frac{dr'}{r'^l}=\frac{2l+1}{(l+2)(l-1)}r-\frac{1}{l-1}\frac{r^l}{R^{l-1}}. \tag{12}$$

Thus, by substituting above equation in Eq. (11) and performing some algebra, we obtain the potential inside the sphere, given by

$$U_d^{\text{in}}(\mathbf{r})=\frac{M_s}{24}\left(9pr-8pR+15r\cos\gamma-16R\cos\gamma+3r\cos2\theta(p-\cos\gamma)\right) \tag{13}$$

which corresponds to Eq. (7) in the main text. Note that the above result coincides with Ref. [1]. Next, the corresponding magnetostatic field reads

$$\mathbf{H}_d^{\mathrm{in}}(\mathbf{r}) = -\nabla U_d^{\mathrm{in}}(\mathbf{r}) = -\frac{M_s}{8}\Big[(3p+5\cos\gamma+(p-\cos\gamma)\cos 2\theta)\,\hat{r}+2\,((-p+\cos\gamma)\sin 2\theta)\,\hat{\theta}\Big], \tag{14}$$

which is Eq. (7) in the main text.

2. Region $r > R$ (outside the sphere)

In this case, it is always satisfied that $r_< = r'$ and $r_> = r$. Therefore, the integral involved in the second term of Eq. (11) is

$$\int_0^R \frac{r_<^l}{r_>^{l+1}} r' dr' = \frac{1}{r^{l+1}} \int_0^R r'^{l+1} dr' = \frac{1}{l+2}\frac{R^{l+2}}{r^{l+1}}. \tag{15}$$

From the substitution of above equation in Eq. (11), we obtain

$$U_d^{\mathrm{out}}(\mathbf{r}) = \frac{M_s(p-\cos\gamma)}{12}\left(3\cos^2\theta - 1\right)\frac{R^4}{r^3}, \tag{16}$$

whose corresponding magnetostatic field is given by

$$\mathbf{H}_d^{\mathrm{out}}(\mathbf{r}) = M_s\frac{R^4}{48r^4}(p-\cos\gamma)\left[\left(\frac{1+3\cos 2\theta}{2}\right)\hat{r}+\sin 2\theta\,\hat{\theta}\right], \tag{17}$$

which correspond to Eq. (8) in the main text.

B. Magnetostatic potential represented in the frame O'

As stated in the main text, the way we chose to explore the magnetostatic interaction between two spheres hosting a BP as a metastable state is by locating a BP_1 in a frame O, which generates a magnetostatic field $\mathbf{H}_{d1}^{\mathrm{out}} = -\nabla U_{\mathrm{d1}}^{\mathrm{out}}$. Then a BP_2, situated in a frame O', interacts with the BP_1 through the integral presented in Eq. 13 of the main text. Therefore, the problem is reduced to express $U_{\mathrm{d1}}^{\mathrm{out}}$ in terms of the O'-variables, i.e., as seen from the frame O'. To calculate the

magnetostatic potential in the frame O', we can transform coordinates from one system to the other as

$$\mathbf{r}-\mathbf{d}=\mathscr{R}\mathbf{r}', \tag{18}$$

where $\mathscr{R}$ is the rotation from the system O to O', and $\mathbf{d}=(d_x,d_y,d_z)$ is a vector pointing from O to O', whose magnitude is the distance between the center of the spheres. Because BPs are axial, we need only two rotation angles to relate the frames with each other. Let's consider the rotation $\mathscr{R}=Y_\psi X_\xi$, rotation of angle ξ around X and then a rotation of ψ around Y. That is

$$\mathscr{R}=\begin{pmatrix}\cos\psi & \sin\xi\sin\psi & \cos\xi\sin\psi\\ 0 & \cos\xi & -\sin\xi\\ -\sin\psi & \cos\psi\sin\xi & \cos\xi\cos\psi\end{pmatrix}. \tag{19}$$

From the above considerations, we have that the magnetostatic potential generated by the BP_1 at the origin of O is

$$U_{\mathrm{d}1}=\frac{c_1}{r_1^3}(3\cos^2\theta_1-1), \tag{20}$$

where $c_1=\frac{M_{s1}}{12}(p_1-\cos\gamma_1)R_1^4$ (see Eq. 16) and the sub-indices $1,2$ are used to referring to frames O and O', respectively. Now, using the relation

$$\mathbf{r}_1=\mathbf{d}+\mathscr{R}\mathbf{r}_2, \tag{21}$$

we can compute the interaction energy at the frame O'. Indeed, the relation of the polar angles between both frames reads

$$\cos\theta_1=\frac{z_1}{r_1}=\frac{d_z+r_2\cos\theta_2\cos\xi\cos\psi+r_2\sin\theta_2(\sin\xi\cos\psi\sin\phi_2-\sin\psi\cos\phi_3)}{|\mathscr{R}\mathbf{r}_2+\mathbf{d}|}, \tag{22}$$

where

$$\mathscr{R}\mathbf{r}_2=\begin{pmatrix}d_x+r_2\cos\phi_2\cos\psi\sin\theta_2+r_2\sin\psi\,(\cos\theta_2\cos\xi+\sin\theta_2\sin\xi\,\sin\phi_2)\\ d_y-r_2\cos\theta_2\sin\xi+r_2\cos\xi\,\sin\theta_2\sin\phi_2\\ d_z+r_2\cos\theta_2\cos\xi\,\cos\psi+r_2\sin\theta_2\,(\cos\psi\sin\xi\,\sin\phi_2-\cos\phi_2\sin\psi)\end{pmatrix}. \tag{23}$$

Therefore, the magnetostatic potential generated by the BP_1 seen from the system 2 (O') becomes

$$U_{\mathrm{d}1} = \frac{c_1}{|\mathscr{R}\mathbf{r}_2 + \mathbf{d}|^3}\left[3\left(\frac{d_z + r_2\cos\theta_2\cos\xi\cos\psi + r_2\sin\theta_2(\sin\xi\cos\psi\sin\phi_2 - \sin\psi\cos\phi_2)}{|\mathscr{R}\mathbf{r}_2 + \mathbf{d}|}\right)^2 - 1\right] \tag{24}$$

from which we can formally calculate the expression for E_{int} in Eq. (13).

[1] Andreas, Ch., Forschungszentrum Jülich, Vol. **88** (2014).
